# Prescriptive appropriateness of dalbavancin in acute bacterial skin and skin structure infections in adults: an integrated approach between clinical profile, patient- and health system-related factors and focus on environmental impact

**DOI:** 10.3389/frabi.2024.1405401

**Published:** 2024-04-24

**Authors:** Francesco Di Gennaro

**Affiliations:** Clinic of Infectious Diseases, Department of Precision and Regenerative Medicine and Ionian Area (DiMePRe-J), University of Bari, Bari, Italy

**Keywords:** dalbavancin, ABSSSIs, pharmacoeconomics, patient compliance, antibiotic therapy, MRSA, clinical efficacy

## 1 Introduction

In the realm of infectious diseases, the management of Acute Bacterial Skin and Skin Structure Infections (ABSSSIs) poses a significant challenge to healthcare providers. Dalbavancin, a long-acting lipoglycopeptide antibiotic, has emerged as a promising option in the treatment of ABSSSIs in adults. This article aimed at exploring the prescriptive appropriateness of dalbavancin by adopting an integrated approach which considered clinical profile, patient- and health system-related factors, and environmental impact.

## 2 Clinical profile: efficacy and safety

Dalbavancin’s pharmacokinetic characteristics, including its prolonged half-life, allow for a weekly dosing schedule, which could be seen as convenient. Its effectiveness against a range of Gram-positive bacteria, such as methicillin-resistant Staphylococcus aureus (MRSA), suggests it might be a viable treatment option (Monteagudo-Martínez et al., 2022). Clinical studies have indicated success rates for dalbavancin between 80% and 98%, comparable to standard treatments (Simonetti et al., 2021; Oliva et al., 2023). Evaluating dalbavancin’s safety is also important, although adverse events have been infrequently reported in the literature, underscoring the importance of ongoing monitoring for any unusual or infrequent side effects (Dunne et al., 2016; Simonetti et al., 2021). Comparatively, its safety profile has been somewhat favorable, especially in terms of renal side effects, which tend to be more common with traditional treatments that require more frequent dosing and longer durations (Dunne et al., 2016).

## 3 Possible adverse events of dalbavancin treatment

While Dalbavancin is recognized for its effective antibacterial activity and favorable dosing schedule, it is essential to consider the spectrum of potential adverse events associated with its use. Among these, allergic reactions warrant particular attention due to their potential to be severe and long-lasting. Allergic reactions to dalbavancin, though relatively rare, can range from mild skin rashes to severe cases of anaphylaxis. The incidence of such reactions has been documented as low but is critically important for clinicians to monitor due to the severity these reactions can entail. For example, hypersensitivity reactions were reported in clinical trials, albeit infrequently (Dunne et al., 2016; Simonetti et al., 2021). Beyond allergic reactions, dalbavancin’s safety profile has been characterized by several other adverse events, though typically with a low incidence. Commonly reported side effects include:

-Nausea and diarrhea, reflecting the most frequently reported gastrointestinal side effects.
-Headaches and dizziness.
-Infusion-related reactions such as flushing, pruritus, and rash at the site of injection.

An important consideration in dalbavancin treatment is its long half-life, which, while advantageous for dosing frequency, means that any adverse reactions, including allergic ones, could potentially persist longer than those associated with antibiotics having shorter half-lives. Therefore, patients receiving dalbavancin should be carefully monitored for adverse reactions (Monteagudo-Martínez et al., 2022).

Given dalbavancin’s extended half-life, clinicians should be aware that any adverse reactions might last longer than those associated with antibiotics with shorter half-lives, necessitating prolonged monitoring (Caselli et al., 2024).

Proper monitoring for adverse reactions is essential, entailing:

- Screening for allergies to dalbavancin or related compounds prior to administration.
- Observing patients for signs of hypersensitivity during and after infusion.
- Educating patients on the importance of reporting any symptoms of allergic reactions promptly (Righi et al., 2022).

Continuous surveillance and patient education remain key components in the clinical use of dalbavancin, ensuring both its effectiveness and safety are maintained (Oliva et al., 2023).

## 4 Health system-related factors

### 4.1 Indirect effects of the setting of care: increased risk of healthcare-associated infections

Beyond the direct impact on patients, the choice of antimicrobial agents for ABSSSI treatment has implications for the broader healthcare system. Outpatient administration of dalbavancin, completely avoiding hospitalization, or its use in de-hospitalizing patients at an early stage can potentially mitigate the risk of the healthcare-associated infections (HAIs) associated with prolonged hospital stays.

A recent meta-analysis has shown almost a 2-fold higher risk of HAIs for patients over 60 years of age with comorbidities, such as diabetes, cardiovascular diseases (also risk factors for ABSSSI), a 6-fold higher risk for those on antibiotic therapy, a 13-fold higher risk for those hospitalized for more than 15 days (Liu et al., 2023).

Therefore, the choice of using Dalbavancin to achieve early de-hospitalization (especially for patients at high risk of HAIs), together with outpatient treatment regimens, can significantly reduce the risk of HAI.

This is also relevant as acquiring a healthcare-related infection increases the risk of death at both 30 days and 1 year, representing a major impact on patient health and life expectancy (Koch et al., 2015). This represents an important aspect from both a clinical and a public health point of view. Considering the increasing importance of infection control, especially in the era of emerging resistant pathogens, the potential of dalbavancin to reduce the burden on inpatient facilities also aligns with the importance of minimizing the indirect consequences of healthcare interventions.

### 4.2 Pharmacoeconomics

In the era of healthcare sustainability, pharmacoeconomic considerations play a pivotal role in decision-making. While dalbavancin may have a higher upfront cost as compared to traditional regimens, its extended dosing interval reduces the need for hospitalization and intravenous access, potentially leading to cost savings in the long run. An in-depth analysis of pharmacoeconomics has shown how a regimen with dalbavancin, compared to the standard of care, has allowed shorter hospital stays, fewer adverse events, and reduced overall costs with significant economic savings while maintaining and, in fact, improving patient outcomes (Poliseno et al., 2021).

### 4.3 Nurse workload and health professional competences

In a historical moment in which human resources in healthcare are quantitatively scarce, their correct allocation is fundamental in improving public health and patient treatment strategies. In Italy for example, there are fewer nurses than the European average (Agenzia Nazionale per i Servizi Sanitari, 2022). Antibiotic strategies, such as long-acting monotherapy strategies which lighten the workload for nurses as compared to multidose strategies must also be evaluated from the perspective of an integrated strategy of healthcare resources. Having few nurses assigned to many beds with multiple therapies can increase the risk of work overload and the consequent risk of healthcare-related or other infections.

On the other hand, the introduction of dalbavancin necessitates a paradigm shift in the roles and competences of healthcare professionals. As an outpatient antibiotic option, healthcare providers must adeptly navigate the complexities of patient education, remote monitoring, and shared decision-making. The integration of dalbavancin into clinical practice requires ongoing training in order to provide healthcare professionals with the knowledge and skills to make informed decisions, thus ensuring optimal patient outcomes and the successful implementation of this novel approach.

## 5 Patient-related factors: compliance and quality of life

The success of any antibiotic outpatient regimen hinges on patient adherence to self-administered drugs. This is crucial especially for vulnerable populations who must adhere to therapeutic regimens, i.e. people with lower socio-economic status or those with a high pill burden for multiple comorbidities.

In fact, as various studies have shown, therapeutic adherence, including antibiotics, is closely related to socio-economic level, making the choice of a long-acting drug an effective strategy for treating patients with a high risk of non-adherence (Mallah et al., 2022) (e.g., poly-comorbidities with a high pill burden and vulnerable populations, such as the homeless and migrants).

Beyond the clinical and economic aspects, the impact of ABSSSI and its treatment on patient quality of life should be acknowledged. The less frequent dosing regimen of dalbavancin not only facilitates better adherence but also allows patients to resume their daily activities more swiftly and their family life. Potential improvement in the quality of life and the impact on adherence should be factored into the overall assessment of the appropriateness of dalbavancin, highlighting the importance of a patient-centric approach.

## 6 Environmental considerations

Addressing climate change transcends beyond the environmental sector, posing a profound challenge to global health. It demands a concerted effort also from healthcare workers a to make informed choices that diminish the ecological footprint, thus ensuring the planet’s sustainability. The healthcare industry, pivotal in safeguarding health and well-being, is paradoxically a significant contributor to carbon emissions, which fuel climate change (Lenzen et al., 2020).

Recent analyses reveal that therapies involving multiple intravenous (IV) administrations are notably more detrimental to the environment, primarily due to their higher carbon dioxide (CO2) emissions when compared with oral or long-acting drug therapies (Tennison et al., 2021; Eii et al., 2023). This distinction stems from the comprehensive lifecycle of IV drugs, which encompasses not only their production and transportation but also the storage and usage phases. Significantly, the environmental toll is exacerbated by the resources expended on IV drug administration accessories, such as syringes, non-sterile or sterile gloves, cannulas, and their dressings (Eii et al., 2023).

A compelling study by Eii et al (Eii et al., 2023). estimates that the carbon footprint associated with the lifecycle of multiple IV antibiotic therapies can be up to 100 times greater than that of alternative therapeutic forms. This disparity highlights the extensive CO2 emissions from the production processes, transportation, and the energy demands of healthcare facilities for storing and administering these therapies, in addition to the waste generated (Eii et al., 2023).

Transitioning towards more environmentally friendly healthcare practices, such as prioritizing oral and long-acting therapies (such as Dalbavancin) where clinically feasible, represents a tangible step towards minimizing the sector’s environmental impact. This strategic shift not only aligns with global sustainability objectives but also underscores the healthcare sector’s commitment to reducing its carbon footprint, all while maintaining high standards of patient care (Vallée, 2024). Moreover, the adoption of sustainable practices, including medical waste recycling and the optimization of drug delivery systems, further contributes to the sector’s environmental stewardship (Berniak-Woźny and Rataj, 2023; Walpole et al., 2023).

Incorporating sustainability into healthcare decision-making processes enables practitioners to navigate the intricate balance between delivering optimal patient care and mitigating the environmental impacts of their practices. It is crucial for the healthcare sector to embrace a holistic view of health that inherently includes environmental sustainability as a fundamental aspect of promoting public health and combating climate change (World Health Organization, 2023).

## 7 Antimicrobial stewardship and integration of approaches using a multidisciplinary approach

In the context of increasing antimicrobial resistance, the role of dalbavancin in antimicrobial stewardship cannot be understated. Its extended dosing interval not only streamlines treatment but also aligns with the principles of responsible antibiotic use. By minimizing unnecessary exposure to antibiotics and to hospital stay which also reduces the risk of resistance development, dalbavancin contributes to the broader efforts aimed at preserving the effectiveness of antimicrobial agents.

Long-acting antibiotic strategies offer a promising avenue for reducing antimicrobial resistance (AMR), based on findings that patients receiving extended intravenous antibiotic treatments for more than 14 days face an increased risk of healthcare-associated infections (HAIs) (Liu et al., 2023). Transitioning to long-acting antibiotic regimens significantly lowers the chance of encountering care-related infections, thereby reducing the need for further antibiotic interventions and, consequently, the potential for AMR development (Micheli et al., 2023).

The decision to prescribe dalbavancin should involve multidisciplinary collaboration, bringing together infectious disease specialists, pharmacists, healthcare economists, and other stakeholders. This collaborative effort ensures that diverse perspectives contribute to the decision-making process, fostering a comprehensive and well-informed approach which considers the nuances of both clinical practice and healthcare economics.

## 8 Conclusions

As the field of infectious disease management changes, the need to consider the effectiveness, patient and health system factors, and environmental impacts becomes increasingly important. This editorial highlights the need for a broad view when assessing dalbavancin’s use for ABSSSI in adults. By looking at these comprehensive aspects, healthcare providers can improve treatment results, make better use of resources, and focus on patient satisfaction in ABSSSI care.

To sum up, deciding on using dalbavancin for ABSSSI requires looking at more than the usual factors. Evaluating its effects on healthcare-associated infections (HAIs), patient needs, environmental concerns, and system challenges, as well as understanding the changing roles of healthcare professionals, is key to adopting a more inclusive and flexible approach to treating ABSSSI in adults. This broader perspective is essential for dealing with the complexities of today’s healthcare and improving outcomes for both patients and the healthcare system as a whole.

## Author contributions

FG: Writing – original draft, Writing – review & editing
